# The *Enterococcus faecalis* Exoproteome: Identification and Temporal Regulation by Fsr

**DOI:** 10.1371/journal.pone.0033450

**Published:** 2012-03-12

**Authors:** Jayendra Shankar, Rachel G. Walker, Deborah Ward, Malcolm J. Horsburgh

**Affiliations:** Institute of Integrative Biology, University of Liverpool, Liverpool, United Kingdom; East Carolina University School of Medicine, United States of America

## Abstract

Analysis of the culture supernatant exoproteins produced by two PFGE clusters of high-level gentamicin and ciprofloxacin-resistant clinical isolates of *Enterococcus faecalis* from the UK and Ireland revealed two distinct protein profiles. This grouping distinguished OG1RF and GelE metalloprotease-expressing isolates from JH2-2 and other GelE-negative isolates. The integrity of the *fsrABDC* operon was found to determine the exoproteome composition, since an *fsrB* mutant of strain OG1RF appeared very similar to that of strain JH2-2, and complementation of the latter with the *fsrABDC* operon produced an OG1RF-like exoproteome. The proteins present in the supernatant fraction of OG1RF were separated using 2D gels and identified by mass spectrometry and comprised many mass and pI variants of the GelE and SprE proteases. In addition cell wall synthesis and cell division proteins were identified. An OG1RF *fsrB* mutant had a distinct exoprotein fraction with an absence of the Fsr-regulated proteases and was characterised by general stress and glycolytic proteins. The exoproteome of the OG1RF *fsrB* mutant resembles that of a *divIVA* mutant of *E. faecalis*, suggestive of a stress phenotype.

## Introduction

Recent years have seen greater study of medically important opportunistic bacterial pathogens due to increased levels of nosocomial infection and antibiotic resistance [1], [2]. Of these pathogens, *Enterococcus faecalis* is prominent due to the frequency of disease and its implication in antibiotic resistance transfer to *Staphylococcus aureus* and *Listeria* species [3]–[6].

The pathogenesis of *E. faecalis* infections is relatively poorly understood; however, its interaction with the environment via secreted exoproteins and surface-attached proteins facilitate colonisation and pathology [7]–[9]. Exoprotein virulence factors of *E. faecalis* identified to date include cytolysin, gelatinase (GelE) and serine protease (SprE). Transcription of the *gelE*-*sprE* operon is temporally regulated via FsrA, the response regulator of the density-dependent two-component system. Insertional inactivation of this locus ablates expression of these proteases and the Fsr system is the only known regulatory locus for the genes [10]. In addition to the prominent virulence exoproteins, GelE and SprE, the Fsr system is a global regulator of an array of surface-expressed and metabolic proteins. Microarray analysis of an *fsrB* mutant identified 119 upregulated and 323 downregulated genes in the later stages of growth [11]. The *fsrABDC* locus is similar to the *agrBDCA* locus of *S. aureus*, but it lacks the RNAIII riboregulator and transcriptional activation appears to function solely via FsrA [10]–[13]. Density-dependent activation of the Fsr system occurs via extracellular accumulation of gelatinase biosynthesis activating pheromone (GBAP, the product of *fsrD*) [14].

The frequency of the *gelE-sprE* operon among isolates was reported to be 93% of 152 clinical isolates [13]; however, the activity of GelE may not be as high as this would suggest due to variation in the frequency of the *fsr* locus. Within clinical isolates from urine, 69% lacked GelE activity but 88% carried the gene [14]. Nakayama *et al.* [14] reported a 23.9 kb deletion, which included the genes *fsrA*, *fsrB* and part of *fsrC*, in 91% of *gelE*-positive strains lacking GelE activity. More recently, Galloway-Pena *et al.* [15] analysed genome sequences of 22 *E. faecalis* strains and identified three major groups based on a highly variable region between *fsrC* (EF_1820) and EF_1841, with the 23.9 kb deletion accounting almost exclusively for an absence of GelE expression [15].

The exoprotein fraction represents a secreted pool of proteins that can interact with the host in commensalism and disease. In *S. aureus* and *Streptococcus (Strep) pyogenes*, this protein fraction contains toxins, enzymes and immune modulators [16]–[17]. This exoprotein fraction was therefore examined by 2D SDS-PAGE in order to identify putative novel virulence factors. Given the demonstrated regulatory role of the *fsr* locus, its contribution together with *gelE* to the regulation of exoproteins produced by *E. faecalis* OG1RF were analysed by using isogenic mutants. Based on exoprotein profiles, 47 clinical strains were analysed and categorised as either OG1RF- or JH2-2-like, indicating GelE activity. However, not all strains that lacked GelE activity contained the 23.9 kb deletion in the *fsr* locus, indicating that other factors may be involved in the regulation of this virulence factor, such as point mutations in *fsr* described recently [18].

## Methods

### Bacterial growth and DNA amplification

Strains and plasmids used in this study are listed in Table 1. Bacterial strains were cultured in BHI (Merck) or LB (Lab M) broth for *E. faecalis* and *Escherichia* (*Esch.*) *coli*, respectively. For standard growth, an overnight broth culture of *E. faecalis* was diluted 100-fold dilution into 50 ml fresh BHI in a 250 ml flask, and incubated with shaking at 250 r.p.m. at 37°C. Antibiotics were included in overnight cultures where appropriate at the following concentrations: 2 mg ml^−1^ kanamycin, 10 µg ml^−1^ erythromycin for *E. faecalis* and 100 µg ml^−1^ ampicillin for *Esch. coli*. To determine protease activity, *E. faecalis* strains were inoculated on BHI agar containing 5% (w/v) dried skimmed milk powder. Following overnight growth at 37°C, plates were examined for zones of clearing, which indicated protease activity by the strains. *E. faecalis* was transformed as described previously [11]. Amplification of the *fsr* locus by PCR was done using primers for *fsrAB* (PR21: 5′CGGTAAGCTCACAGAAG and PR22: 5′GGCAGGATTTGAGGTGC) or EF_1841-*fsrC* described previously [14].

**Table 1:** *E. faecalis* strains used in this study.

*E. faecalis* Strain	Alternative ID	Characteristics	Source
OG1RF		Wild type Rif ^r^/Fus^r^	[42]
JH2-2		Wild type Rif ^r^/Fus^r^ naturally occurring *fsr* deficient strain	[45]
OG1RF *gelE*	TX5264	In-frame deletion of *gelE*	[43]
OG1RF *fsrB*	TX5266	*fsrB* deletion mutant	[13]
OG1RF *fsrB* pTEX5249	TX5245	*fsrB* deletion mutant with *fsrABDC* complementation on pTEX5249	[13]
JH2-2 pTEX5249	LIV305	*fsr* deficient strain with *fsrABDC* complementation on pTEX5249	This study
V583		Van^r^	[44]
EC23, EC117, EC95, EC207, EC238		PFGE cluster 1 BSAC Bacteraemia Resistance Surveillance Programme (UK & Ireland)	[24]
EC126, EC137, EC36, EC127, EC216		PFGE cluster 2 BSAC Bacteraemia Resistance Surveillance Programme (UK & Ireland)	[24]
402,463,486,487, 488,489,499 (/96/NIPH)		Clinical isolates (Poland)	[30]
IS19-IS48		MLST-typed clinical isolates(Spain)	[26]

### Exoprotein extraction for 1D PAGE

*E. faecalis* strains were grown overnight in BHI broth, then subcultured for the desired time period before removing aliquots and pelleting cells by centrifugation. TCA was then added to the supernatant at 10% (w/v) final concentration and incubated for 30 minutes on ice. Precipitated proteins were recovered by centrifugation and the protein pellet was washed with buffered wash solution [50 mM Tris-HCl pH 6.8, 95% (v/v) ethanol]. The final protein pellet was resuspended in Laemmli loading buffer [19] prior to loading on an 11% (w/v) separation gel. After separation, the gels were stained with Coomassie stain [25% (v/v) isopropanol, 10% (v/v) acetic acid, 0.25% (w/v) Coomassie R-250] for 30 min and destained [10% (v/v) methanol, 10% (v/v) acetic acid] overnight.

### Exoprotein extraction and 2D SDS-PAGE

Proteins were precipitated from cell culture supernatants using previously described method for *Strep. pyogenes* [20] . Briefly, cells were grown to the required growth phase in dialysed BHI medium and harvested. The cell culture supernatant was filtered (0.22 µm membrane) before precipitating proteins on ice for 60 min by adding TCA∶acetone [10% (w/v)∶5% (v/v)]. Proteins were pelleted by centrifugation and the pellet was washed [50 mM Tris-HCl pH 6.8, 95% (v/v) ethanol] and air-dried. The protein pellet was resuspended in 50 mM Tris-HCl (pH 7.5) containing DNase and RNase (both 10 µg ml^−1^) and incubated at 37°C for 15 min. Exoprotein purification for *E. faecalis* OG1RF *fsrB* was described in [21]. Proteins were precipitated and washed once more before proteins were resuspended in 150 µl 2D lysis buffer [7 M urea, 2 M thiourea, 4% (w/v) CHAPS, 1% (w/v) DTT, 1 mg bromophenol blue] and stored at −80°C until use.

Soluble protein (500 µg) was brought up to 320 µl with rehydration buffer [8 M urea, 2 M thiourea, 4% (w/v) CHAPS, 20 mM DTT, 1% (v/v) ASB 14 detergent and 0.5% (v/v) carrier ampholytes (Bio-lyte 3/10, Bio-Rad)]. Samples were kept for 1 h at room temperature with gentle shaking. Samples were in-gel rehydrated and focused on 11 cm, pH 4–7 IPG strips (Bio-Rad) for a total of 40,000 V h^−1^ [150 V for 1 h, 300 V for 1 h, 600 V for 1 h, 1,200 V for 1 h, 1,200–8,000 V over 1 h (linear gradient), 8,000 V to 40,000 V (steady state)], using a Protean IEF Cell (Bio-Rad). After focusing, strips were equilibrated in 50 mM Tris (pH 6.8), 6 M urea, 2% (w/v) SDS, 30% (w/v) glycerol and bromophenol blue, containing 20 mM DTT in the reduction step (15 min) and 25 mM iodoacetamide in the alkylation step (15 min). IPG strips were run in the second dimension on 16×16 cm 12.5% SDS-PAGE gels using a Protean II xi 2D Cell (Bio-Rad). Gels were run in triplicate, silver-stained [22] and scanned (GS-710 Densitometer, Bio-Rad) as gray-scale TIFF files at 16 bit and 300 dpi and uploaded into the Progenesis ‘SameSpots’ (Non Linear Dynamics) gel image analysis Software. Quantitative analysis was based on average gels created from three gel replicates. Spots in the treated samples with a *P* value≤0.05 and greater than twofold difference from the control sample were considered statistically significant. For protein identification by mass spectrometry, two gels containing 500 µg each of soluble protein were prepared as above and stained with Colloidal Coomassie Brilliant Blue [23]. Scanned images were uploaded into Progenesis ‘SameSpots’ and matched to the analytical gels.

### Protein identification

Spots for identification were excised and digested in-gel with trypsin. Gel plugs were destained with 50% (v/v) acetonitrile in 50 mM ammonium bicarbonate, dehydrated in 100% acetonitrile and rehydrated overnight in 10 µl 50 mM ammonium bicarbonate containing 20 ng µl^−1^ MS grade trypsin (Promega), all at 37°C.

Peptide mass fingerprints (PMFs) were generated by using a reflectron MALDI-TOF instrument (M@LDI; Waters-Micromass). Supernatants (above) were mixed (1∶1 ratio) with a saturated solution of α-cyano-4-hydroxycinnamic acid ACN∶water∶TFA [50∶49∶1 (v/v/v)]. The acquired spectra were analysed using MassLynx v4.0 (Waters-Micromass) and were all externally calibrated with a mixture of peptides. For each sample, all acquired spectra were combined and processed as follows using MassLynx v 4.0: smoothing, 2× smooth using a Savitzky Golay method set at ±3 channels and background subtraction using a polynomial of order 1 and 40% below the curve in order to reduce background noise. To get accurate mono-isotopic peak data all processed spectra were centred using the top 80% of each peak. Peak lists were generated by using ProteinLynx, part of MassLynx v 4.0. Monoisotopic peptide masses in the mass range of 800–4000 Da were used in the database search. The resulting peptide mass maps were used to interrogate Firmicute sequences to generate statistically significant candidate identifications using the MASCOT search engine (http://www.matrixscience.com). Searches were performed allowing for complete carbamidomethylation (alkylation) of cysteine residues, partial oxidation of methionine residues, one missed cleavage and a mass error of ±0.5 Da. Molecular weight search (MOWSE) scores, number of matched ions, percent protein sequence coverage and correlation of gel region with predicted mass and pI were collectively considered for each protein identification. Inter-genome protein comparisons were performed using SEED-Viewer (http://www.theseed.org).

## Results

### Distinct exoprotein patterns in *E. faecalis* strains

**Figure 1:**
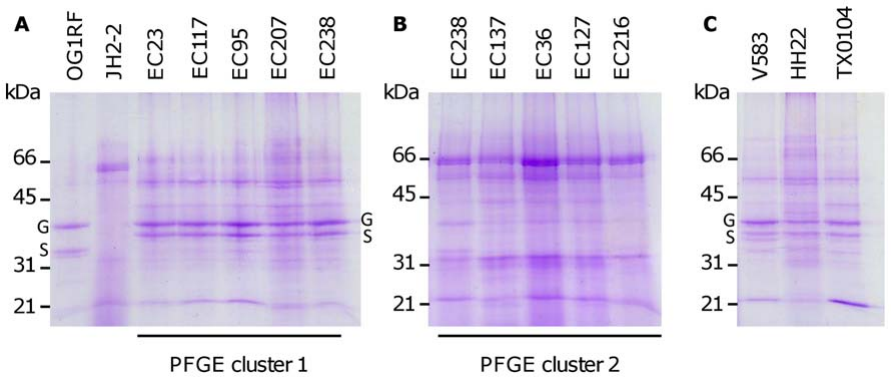
Exoproteins from *E. faecalis* strains OG1RF and JH2-2 and clinical isolates from separate PFGE clusters. Stationary phase (8 h) culture supernatant from (A) strains OG1RF, JH2-2, PFGE cluster 1 strains EC23, EC117, EC95, EC207, EC238. (B) PFGE cluster 2 strains EC126, EC137, EC36, EC127, EC216. (C) strains V583, HH22, TX0104. GelE (G) and SprE (S) were identified by mass spectrometry. Molecular mass markers are indicated (kDa).

Stationary phase exoproteins were purified from culture supernatants of clinical isolates bearing high level gentamicin and ciprofloxacin resistance that were previously divided into two separate PFGE clusters [24]. Culture supernatant exoproteins were separated using SDS-PAGE and compared with those of the commonly used *E. faecalis* laboratory strains OG1RF and JH2-2. Strikingly, two distinct patterns of proteins were observed that separately grouped each PFGE cluster with the protein profile of either strain OG1RF or JH2-2 (Figure 1 A,B). In addition, a comparison with the exoproteins from several genome-sequenced strains of *E. faecalis* (V583, HH22 and TX0104) [25] identified OG1RF-like exoprotein expression patterns (Figure 1C). In contrast with OG1RF, strain JH2-2 is negative for gelatinase activity (Zn-metalloprotease) [26] and consequently the clinical isolates were tested for proteolysis on casein agar. The PFGE clusters were discriminated by their ability to hydrolyse casein, with PFGE cluster 2 lacking activity and matching the known phenotype of JH2-2 (data not shown).

Common to exoproteins from strain OG1RF and OG1RF-like strains was the presence of two prominent proteins with molecular masses of approximately 36 and 38 kDa. To discriminate the OG1RF-like and JH2-2-like strains, these two proteins were cut from gels of exoproteins from strain V583, trypsinised and identified from their MALDI-TOF PMFs using the MASCOT database. These PMFs ascribed identities to the 36 kDa protein as serine protease (SprE) (5 peptides, 23% coverage) and the 38 kDa protein as gelatinase (GelE) (5 peptides, 21% coverage). These proteases are well-described exoproteins regulated via the Fsr two-component signal transduction system that mediates quorum sensing [10].

### Fsr-dependent regulation discriminates OG1RF-like and JH2-2-like exoprotein profiles

**Figure 2:**
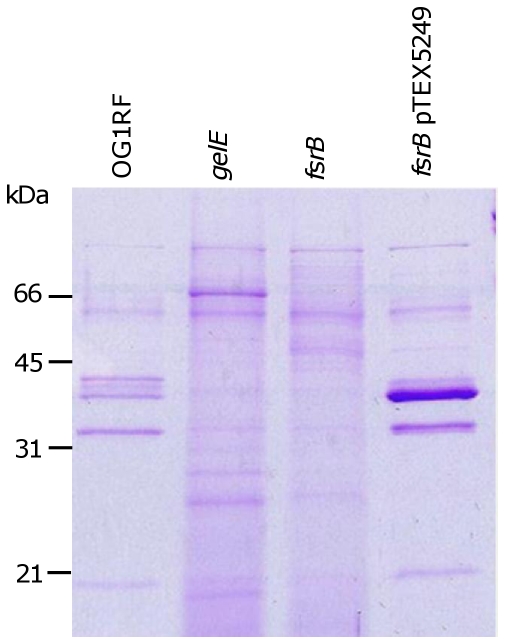
Role of *fsrB* and *gelE* in modulating stationary phase (8 h) culture supernatant exoproteins. *E. faecalis* strains OG1RF and isogenic mutants OG1RF *gelE*, OG1RF *fsrB* and OG1RF *fsrB* pTEX5249. Molecular mass markers are indicated (kDa).

**Figure 3:**
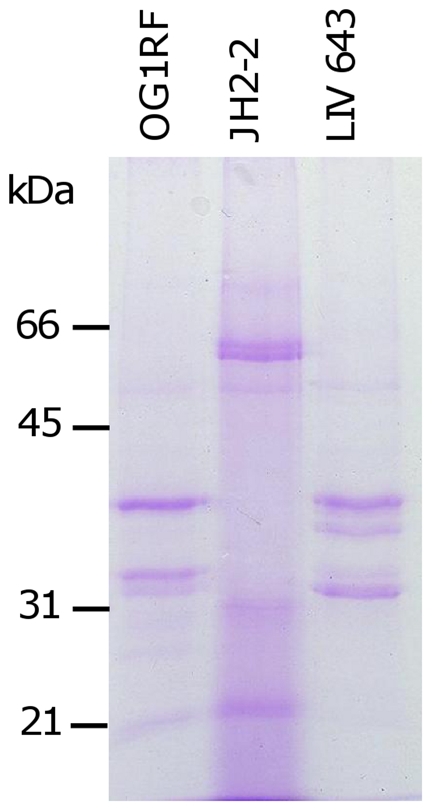
Complementation of Fsr function in strain JH2-2. Stationary phase culture supernatant of strains OG1RF, JH2-2 and LIV643 (JH2-2 pTEX5249). Molecular mass markers are indicated (kDa).

The absence of GelE and SprE proteins from the JH2-2-like strains was proposed here to reflect the known lack of conservation within the *fsrABDC* chromosomal locus [10]–[15]. To confirm the relationship between the observed OG1RF-like exoprotein profile and the presence of the *fsrABDC* operon and GelE, the strains TX5264 (OG1RF *gelE*) and TX5266 (OG1RF *fsrB*) were compared with strains OG1RF and JH2-2 (Figure 2). The association was also investigated by examining the exoproteins of the complementation strain TX5245 (OG1RF *fsrB* pTEX5249) (Figure 2). Inactivation of *fsrB* revealed a marked change to the OG1RF-like exoprotein complement towards that of JH2-2 like strains. A similar, but distinct, exoprotein set was evident with inactivation of *gelE* in strain OG1RF, although this protein complement contains elevated levels of SalB (Shankar *et al*, submitted for publication). In addition, complementation of *fsrB* with the plasmid-encoded *fsrABDC* operon produced overexpression of proteins, likely to represent GelE and SprE (Figure 3). These comparisons revealed that the OG1RF-like exoprotein profile was a signature of a functional *fsrABDC* locus and downstream *gelE-sprE* operon.

**Figure 4:**
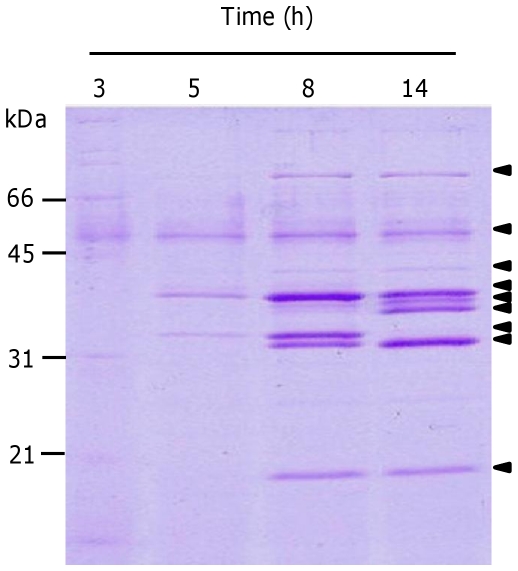
Temporal expression of *E. faecalis* OG1RF exoproteins. Culture supernatant was sampled after early exponential (3 h), mid-exponential (5 h), stationary (8 h) and overnight growth (14 h). Molecular mass markers are indicated (kDa). Arrowheads indicate the 9 most prominent exoprotein bands.

The *fsrABCD* operon-encoded quorum-sensing locus is a known growth-phase regulator of the proteases GelE and SprE [10]–[12]. To identify its contribution to temporal exoprotein expression, culture supernatant of strain OG1RF was sampled at early-exponential (3 h), mid-exponential (5 h) and stationary (8 h) growth phases, together with an overnight (14 h) culture sample. Electrophoretic separation identified clear temporal changes to the exoproteome that consisted of nine major protein bands (Figure 4). It was also confirmed that in contrast with OG1RF, strain TX5266 (OG1RF *fsrB*) revealed an absence of clear temporal changes to the exoproteome (data not shown), supporting the suggestion that Fsr is the sole temporal regulator of the exoproteome in strain OG1RF under the conditions tested.

### Distinct genetic differences produce a JH2-2-like exoprotein pattern of expression

Previous studies identified that deletion of a 23.9 kb region encompassing the majority of the *fsrABCD* operon was the major factor (79% of GelE-negative strains) contributing to a gelatinase-negative phenotype [14]. To determine the nature of the genetic differences producing GelE-negative phenotypes, and thus JH2-2-like exoprotein phenotypes, two separate loci were amplified by PCR; these were: EF1841-*fsrC* and *fsrAB*, and these were amplified from 47 european clinical isolates from the UK [23], Spain [25] and Poland [26] (Table 1), with strains OG1RF and JH2-2 representing *fsr-*positive and negative strains, respectively. JH2-2-like strains were determined by confirming an absence of gelatinase activity on casein agar. Analysis of the clinical isolates indicated four distinct groups: Group 1 strains were GelE-positive and PCR-positive for *fsrAB* and EF_1841-EF_1820 (*fsrC*) (27 isolates); Group 2 strains were GelE-negative and PCR-negative for *fsrAB* and EF_1841- *fsrC* (11 isolates); Group 3 strains were GelE-negative and PCR-negative for *fsrAB* but PCR positive for EF_1841-*fsrC* (10 isolates); Group 4 strains were GelE-negative and PCR-positive for *fsrAB* and EF_1841-*fsrC* (one isolate) (data not shown). These groups of strains indicate that the GelE-negative exoprotein phenotype derives from a diverse set of genetic causes, mostly relating to the *fsr* locus.

### Proteases GelE and SprE are the dominant exoproteins of *E. faecalis* OG1RF

**Figure 5:**
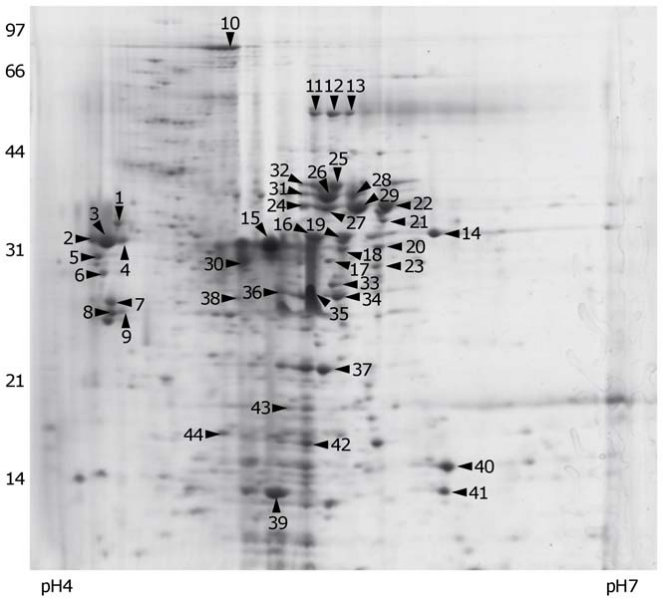
Stationary phase (8 h) exoproteins of *E. faecalis* OG1RF separated by 2D SDS-PAGE. Arrowheads indicate spots to which an identity was assigned after MALDI-TOF analysis. The identified protein spots are listed in Table 2. Molecular mass markers are shown to the left (kDa). A representative image is shown.

The exoproteome of *E. faecalis* OG1RF was purified from cell culture supernatants after 8 h growth (representing early stationary phase) and 500 µg protein was separated by using 2D SDS-PAGE (Figure 5). Protein spots of interest were excised and trypsinised and the masses of component peptides were determined by using MALDI-TOF mass spectrometry. PMFs were queried against the MASCOT database and all protein identifications were assigned from the best matches. A list of identified proteins is given in Table 2. Fifty-nine protein spots were excised from the gels, which yielded 44 protein identities matched to *E. faecalis* V583, with 28 unique identities. Nine protein spots were identified as GelE and seven protein spots were identified as SprE where these existed with several distinct molecular masses and pI values, indicative of post-translational modifications such as charge changes.

**Table 2:** Identification of exoproteins of *E. faecalis* OG1RF.

Spot	Protein description (Gene)	Calculated mass (kDa)/pI	Peptides matched	Coverage (%)
1	GTP-binding protein LepA (EF_2352)	68.27/4.99	10/28	20
2	D-alanine–D-lactate ligase (EF_2294)	21.24/4.7	6/31	52
3	GTP-binding protein LepA (EF_2352)	68.27/4.99	5/11	16
4	DNA primase (EF_1521)	73.01/5.12	6/20	19
5	DNA polymerase III subunit alpha (EF_1044)	31.35/4.96	5/17	35
6	Transcriptional regulator, Cro/CI family (EF_2508)	20.92/5.83	4/18	34
7	VanXYG2; D-alanyl-D-alanine carboxypeptidase (Q30BF0_ENTFA)	29.43/5.21	9/93	55
8	Acetyltransferase, GNAT family (EF_1296)	21.98/5.48	4/18	44
9	Alpha-glycerophosphate oxidase (EF_1928)	67.60/4.94	5/10	16
10	Glycosyl hydrolase, family 20 (EF_0114)	94.12/4.96	11/21	18
11	Lipoteichoic acid synthase, LtaS (EF_1264)	79.96/6.2	8/32	21
12	Hypothetical protein (EF_1995)	12.32/4.29	6/27	60
13	LtaS (EF_1264)	79.96/6.2	8/27	19
14	Endo-beta-N-acetylglucosaminidase (EF_2863)	34.54/5.96	7/29	26
15	Serine protease, SprE (EF_1817)	31.04/5.54	16/105	42
16	Serine protease, SprE (EF_1817)	31.04/5.54	14/56	39
17	Septation ring formation regulator EzrA (EF_0370)	68.11/4.75	8/27	24
18	Serine protease, SprE (EF_1817)	31.04/5.54	9/31	38
19	Serine protease, SprE (EF_1817)	31.04/5.54	5/19	25
20	Serine protease, SprE (EF_1817)	31.04/5.54	15/82	57
21	Zn-Metalloprotease, GelE (EF_1818)	55.34/4.99	10/33	32
22	GelE (EF_1818)	55.34/4.99	5/13	15
23	Transcriptional regulator, TetR family (EF_1531)	20.70/8.63	4/21	48
24	GelE (EF_1818)	55.34/4.99	6/16	14
25	D-alanyl-D-alanine dipeptidase (EF_2293)	23.20/5.8	4/16	26
26	Zn-Metalloprotease, GelE (EF_1818)	55.34/4.99	6/13	16
27	Zn-Metalloprotease, GelE (EF_1818)	55.34/4.99	6/18	16
28	Zn-Metalloprotease, GelE (EF_1818)	55.34/4.99	7/16	19
29	Zn-Metalloprotease, GelE (EF_1818)	55.34/4.99	10/34	32
30	Serine protease, SprE (EF_1817)	31.04/5.54	12/71	50
31	Zn-Metalloprotease, GelE (EF_1818)	55.34/4.99	7/21	19
32	Zn-Metalloprotease, GelE (EF_1818)	55.34/4.99	8/16	23
33	Hypothetical protein (EF_0841)	39.46/5.61	6/58	27
34	DNA replication protein, putative (EF_1279)	29.74/6.19	5/17	31
35	Serine protease, SprE (EF_1817)	31.04/5.54	10/57	39
36	Septation ring formation regulator EzrA (EF_0370)	68.28/4.75	9/20	31
37	Ribosomal protein L11 RplK (EF_2719)	14.68/9.52	5/23	47
39	Hypothetical protein (EF_0486)	8.62/6.23	4/26	66
40	Hypothetical protein (EF_2308)	3.23/5.91	2/15	84
41	Transcriptional regulator, Cro/CI family (EF_2508)	20.92/5.83	5/27	37
42	Hypothetical protein (EF_2843)	8.41/4.6	4/21	56
43	Hypothetical protein (EF_1926)	20.78/9.32	5/25	34
44	ATP synthase F0, AtpE (EF_2613)	7.61/6.07	4/20	63

Spot numbers correspond to those in Figure 5.

All protein matches were also analysed by using SignalP v3.0 (http://www.cbs.dtu.dk/services/SignalP/) [27] to identify potential signal peptide signatures for protein export [28]. PSortb v3.0 (http://www.psort.org/psortb/) [29] was used to determine the most probable localisation of the proteins. Four proteins displayed a signal peptide: GelE [VAA-EE], SprE [AWA-EE], EF_1264 (LtaS) [AYA-VD] and EF_2863 (endo-ß-*N*-glucosaminidase) [VQA-AS].

### The exoproteome of OG1RF *fsrB* is dominated by metabolism- and stress-related proteins

**Figure 6:**
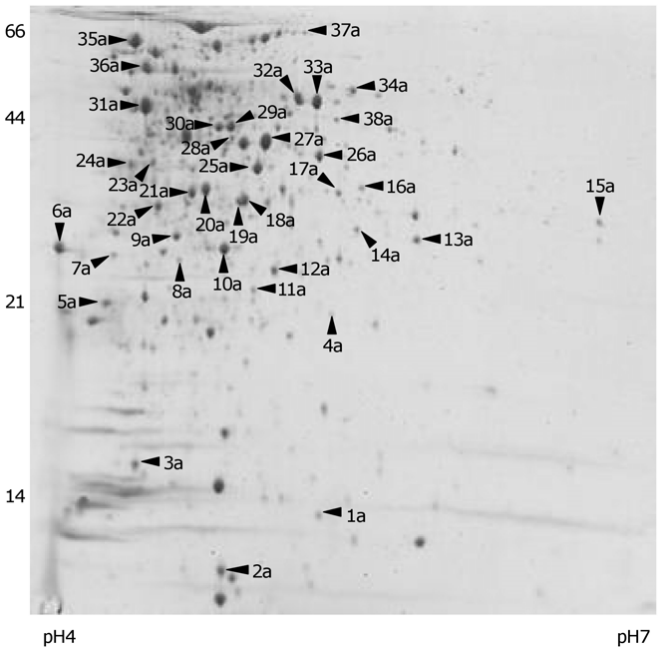
Exoproteins of *E. faecalis* strain TX5266 (OG1RF *fsrB*) following overnight growth separated by 2D SDS-PAGE. Arrowheads indicate spots to which an identity was assigned after MALDI-TOF analysis. Identified protein spots are listed in Table 3. Molecular mass markers are shown (kDa). A representative image is shown.

Analysis of the exoproteome of strain TX5266 (OG1RF *fsrB*) revealed many minor proteins and this protein pattern appeared to be constant at 5 h (exponential phase, data not shown), 8 h (figure 3) and 14 h (overnight, data not shown). To characterise this fraction in more detail, and determine the contributing proteins, a 500 µg sample was separated by 2D-gel electrophoresis (Figure 6). The exoprotein fraction differed from strain OG1RF due to the greater abundance of high molecular weight, low pI proteins. The major protein identities associated with strain TX5266 (OG1RF *fsrB*) are listed in Table 3. A key difference between the two exoproteomes is the absence of the GelE and SprE proteases in the *fsrB* mutant, which dominate the strain OG1RF supernatant fraction. In addition low pI proteins constitute the majority of the exoproteins of strain TX5266 (OG1RF *fsrB*) including metabolism enzymes (e.g. Eno, Pgk, GpmA, Gap-2) and general stress proteins (DnaK, GroEL, AhpC). The protein complement in the culture supernatant did not reflect that which would be expected if this fraction was generated solely by cell lysis, due to the absence of the known dominant intracellular proteins, at least within the spots identified here.

**Table 3:** Identification of exoproteins of *E. faecalis* strain TX5266 (OG1RF *fsrB*).

Spot	Protein description (Gene)	Calculated mass (kDa)/pI	Peptides matched	Coverage (%)
1a	RNA polymerase omega subunit, RpoZ (EF_3126)	11.53/5.36	6/35	51
2a	30S ribosomal protein S6, RpsF (EF_0007)	11.59/5.01	7/11	48
3a	Dps Family protein (EF_3233)	17.93/4.56	6/11	33
4a	Fumarate reductase subunit (EF_2556)	53.83/5.26	12/35	25
5a	Alkyl hydroperoxide reductase, AhpC (EF_2739)	21.3/4.5	16/22	43
6a	N-acetylmuramoyl-L-alanine amidase (EF_1823)	30.32/4.89	3/4	16
7a	Hypothetical protein (EF_1470)	14.62/5.61	5/26	38
8a	ABC transporter, ATP-binding protein (EF_2394)	28.40/4.73	9/13	37
9a	Glycine betaine/carnitine/choline ABC transporter (EF_0863)	34.72/5.14	16/21	41
10a	Fructose-bisphosphate aldolase class II, Fba (EF_1167)	31.02/4.86	7/14	26
11a	Adenylate kinase (EF_0228)	24.25/5.05	9/15	36
12a	Phosphoglycerate mutase I, GpmA (EF_0195)	26.05/5.09	11/21	34
13a	Endo-beta-N-acetylglucosaminidase (EF_2863)	35.54/5.96	6/10	25
14a	Tail protein, putative (EF_1829)	37.97/5.27	8/21	27
15a	Hypothetical protein (EF_0375)	35.43/7.07	9/19	28
16a	Endolysin. Ply-1 (EF_1293)	40.14/5.70	11./1	30
17a	Catabolite control protein A, CcpA (EF_1741)	36.19/5.27	13/14	30
18a	Chitinase, family 2 (EF_0361)	38.31/5.31	6/10	13
19a	Phosphotransacetylase Pta (EF_0949)	35.59/4.97	7/19	30
20a	Translation elongation factor, Ts, Tsf (EF_2397)	32.11/4.87	18/43	51
21a	L-lactate dehydrogenase Ldh (EF_0255)	35.52/4.77	14/38	47
22a	Pyruvate dehydrogenase E1 component, PdhB (EF_1354)	35.37/4.67	12/46	37
23a	Basic membrane protein family (EF_0177)	37.78/4.88	8/15	21
24a	Basic membrane protein family (EF_0177)	37.78/4.88	4/15	14
25a	Arginine deiminase, ArcB (EF_0105)	38.13/5.02	19/28	46
26a	Pyruvate dehydrogenase complex E1 component, PdhA (EF_1353)	41.35/5.25	8/12	24
27a	Glyceraldehyde 3-phosphate dehydrogenase, Gap-2 (EF_1353)	35.92/5.03	7/17	24
28a	Acetate kinase, AckA (EF_1983)	43.49/4.96	12/15	33
29a	Phosphoglycerate kinase, Pgk (EF1963)	42.42/4.90	16/36	49
30a	Arginine deiminase, ArcB (EF_0105)	38.13/5.02	20/26	41
31a	Enolase, Eno (EF_1961)	46.48/4.56	12/34	36
32a	Lipoteichoic acid synthase, LtaS (EF_1264)	79.96/6.2	12/24	15
33a	Lipoteichoic acid synthase, LtaS (EF_1264)	79.96/6.2	19/36	30
34a	ErfK/YbiS/YcfS/YnhG family protein, putative (EF_2860)	52.77/6.74	20/38	35
35a	DnaK (EF_1308)	65.54/4.59	12/20	23
36a	GroEL (EF_2633)	55.41/4.66	18/24	28
37a	Putative ATPase, CbiA (Q9AL20)	34.43/5.17	7/25	25
38a	Glutamate dehydrogenase, GdhA (EF1415)	49.62/5.42	11/33	29

Spot numbers correspond to those in Figure 6.

## Discussion

Analysis of the culture supernatant exoproteins produced by two PFGE clusters of high level gentamicin- and ciprofloxacin-resistant clinical isolates of *E. faecalis* from the UK and Ireland [24] revealed two distinct protein profiles. When compared to the commonly used laboratory strains OG1RF and JH2-2, there was an observed correlation of the exoprotein profiles produced by isolates of these distinct PFGE clusters corresponding to either an OG1RF-like or JH2-2-like exoprotein complement. Since OG1RF and JH2-2 are known to differ in their expression of the virulence-associated Zn-metalloprotease, gelatinase (GelE) [10], [13], [14], the PFGE cluster strains were tested for casein hydrolysis. OG1RF-like strains were all positive for gelatinase activity and revealed the presence of characteristic prominent protein bands of between ∼30 and 40 kDa using 1D SDS-PAGE, which were identified as being GelE and the serine protease SprE.

Previous studies have clearly demonstrated the genetic variance of the *fsrABDC-gelE-sprE* locus resulting from deletion of the 23.9 kb EF_1841-*fsrC* region and have demonstrated the correlation between this deletion and the lack of gelatinase activity [10], [11], [13], [15]. More recently, a larger study testing a diverse set of multilocus sequence types (MLSTs) demonstrated that the EF_1841-*fsrC* deletion was highly conserved with common single nucleotide polymorphisms (SNPs) and junction sequences in strains bearing the deletion; its association with the absence of gelatinase expression was shown to be independent of genetic lineage [15].

In this study, using a set of 47 *E. faecalis* isolates from the UK, Spain and Poland, GelE-negative strains were identified by the absence of casein hydrolysis and then tested by PCR amplification to investigate the basis for the JH2-2-like exoprotein phenotype. Three groups of GelE-negative strain genotypes were discriminated by the presence/absence of the 23.9 kb EF_1841-*fsrC* region and *fsrAB*, indicating that in 21 of 22 isolates the absence of regulatory genes explained loss of expression. Thus, *fsr* locus variants can produce a JH2-2-like exoprotein supernatant fraction. Recent studies [15] clearly demonstrate that the lack of GelE activity in isolates was almost completely due to variant *fsr* locus deletions from an analysis of 22 *E. faecalis* genome sequences. Consequently, the underlying genetic cause for the JH2-2-like exoprotein profile was not unambiguously determined here. The finding in this study that the PFGE clusters identified within the high-level gentamicin and ciprofloxacin bacteraemia strains from the UK could also be grouped by their exoprotein profile was tested with ten isolates. Analysis of MLSTs and gelatinase expression [15] suggests that the relationship between genetic lineage and gelatinase expression would not be maintained in a larger set of strains. The precise mechanistic explanation for this deletion and its spread across different MLSTs requires further study.

Temporal regulation of the exoproteome was demonstrated to be dependent on the *fsr* locus in strain OG1RF, but strain JH2-2, which has the 23.9 kb EF_1841-*fsrC* deletion [14] maintains an apparently constant exoproteome throughout growth in the conditions studied. This *fsr* locus deletion was complemented by transformation with pTEX5249, which contains the *fsrABDC* operon, and temporal expression was restored with a protein complement similar to that of OG1RF. This indicates that *fsr* function, and thus an OG1RF-like exoproteome, can be regained in this naturally *fsr*-deficient strain and strains in the environment could have functionality restored via chromosomal transfer or temperate bacteriophage-mediated generalised transduction [30], [31].

GelE was observed on 2D gels within the 31–44 kDa region. Significant processing of GelE via C-terminal proteolytic cleavage is required for its activation [32] and the presence of GelE spots in this region with minor variations in the pI, indicates post-translational processing of GelE such as charge changes. SprE was identified from seven spots on 2D SDS-PAGE gels, which could be due to GelE-dependent proteolysis or SprE-catalysed self-cleavage [33]. Different isoforms of SprE were previously demonstrated to have different activities [33] and like GelE, pI variants of SprE were observed in this study.

Comparison of the exoproteomes of OG1RF and an isogenic *fsrB* mutant separated by 2D electrophoresis revealed the absence of the cell density-regulated proteases, GelE and SprE. The proteases are predominant in the OG1RF proteome with the remaining proteins identified comprising: cell wall synthesis/modification enzymes lipoteichoic acid synthase (LtaS), endo-b-N-acetylglucosaminidase; cell division proteins (EzrA); and several proteins not clearly assigned to cellular pathways. LtaS (EF_1264) is a transmembrane protein required for the production of the PGP backbone chain of LTA. In *S. aureus*, proteolytic cleavage by the signal peptidase SpsB irreversibly inactivates LtaS during growth [34] and its presence in the exoprotein fraction in the *E. faecalis* OG1RF exoproteome could reflect similar turnover. Whether the released, cleaved protein which contains a predicted sulphatase superfamily domain contributes an alternative function that benefits the cell, such as mucin desulfation, is not known. The OG1RF exoproteome is apparently limited in potential virulence-enhancing determinants. The strain is atypical among genome-sequenced isolates since it is a CRISPR CAS-bearing strain with minimal evidence of horizontal gene acquisition [35]. Comparison of the exoproteome with other clinical isolates is likely to reveal a more extensive variable complement of proteins in *E. faecalis*. Together with the expected absence of the GelE and SprE proteases, the 2D exoproteome of the *fsrB* mutant comprised many glycolytic and general stress proteins. Comprehensive studies of the *S. aureus* exoproteome [21], [36] have identified many of the proteins revealed in the *E. faecalis* exoproteome, suggesting that many of these proteins with defined intracellular functions contribute to the exoproteome. Moreover, some of these have been identified as having extracellular functions e.g. enolase in staphylococci, streptococci and lactobacilli [37]. The *E. faecalis* TX5266 (OG1RF *fsrB*) exoproteome contains many proteins with identifiable secretion signals supporting their extracellular location. This exoproteome was more extensive as judged by the number of spots and their diversity of functions. The wild-type and *fsrB* mutant exoproteomes share few proteins in common, at least with respect to those identified here, and consequently they were not quantitatively compared. The OG1RF *fsrB* mutant was previously described as having an autolysis defect [38] and we have confirmed this finding and shown that the strain has increased dead cells during culture (Shankar *et al*, submitted for publication). Despite the fact that protein spot identification was not exhaustive in this study it is apparent that many of the proteins match those of a *divIVA* mutant [39] and *salB* mutant (Shankar *et al*, submitted for publication). This overlap requires further investigation. The mechanisms underpinning the Fsr-dependent changes in the exoproteome are unclear, but could result from differences in protease expression, cell surface and autolysis changes in the mutant, differences in viability, or a combination of these factors. The OG1RF *fsrB* mutant exoproteome differs from that of the *gelE* mutant due to the presence of SalB in the *gelE* mutant fraction (Fig. 2A). The reason for this visibly pronounced difference is unclear but it likely reflects FsrA-dependent transcriptional differences between the strains, either directly or indirectly.

Recent protein fractionation studies have begun to examine the secreted proteins of *E. faecalis*. Several studies have reported efforts to catalogue and characterise the surface-associated and membrane proteins. Analysis of the surface-associated protein fraction described recently [40] reveals overlap with several of the proteins identified here (e.g. glycolytic, general stress proteins). Exoproteins potentially interact with the environment in several ways via release from the cell and/or via binding back to the cell surface.

The recent and ongoing explosion of genome sequencing in the enterococci will enable comparative *in silico* analyses to determine the pool of potential secreted proteins and several studies have begun to address this [40], [41]. The variability of exoproteins among strains will be dramatic mainly due to the high frequency of those bearing a deletion of the *fsr* locus; however, the variance of exoproteins among *fsr*-replete strains will be important and this study will facilitate this comparative approach.
